# Ambra1 induces autophagy and desensitizes human prostate cancer cells to cisplatin

**DOI:** 10.1042/BSR20170770

**Published:** 2019-08-23

**Authors:** Jie Liu, Zhiyuan Chen, Jia Guo, Lei Wang, Xiuheng Liu

**Affiliations:** 1 Department of Urology, Renmin Hospital of Wuhan University, Wuhan 430060, Hubei Province, China; 2 Department of Urology, Inner Mongolia People’s Hospital, Hohhot 010017, China

**Keywords:** Ambra1, Apoptosis, Autophagy, Cisplatin, Prostate cancer

## Abstract

Prostate cancer (PCa), the second most mortal cancer from developed countries, presents a high level of chemoresistance. There is emerging evidence underscores the critical role of autophagy in the onset, progression, and chemoresistance of PCa. In the present study, we investigated the possible role of a novel autophagy regulator, activating molecule in beclin1-regulated autophagy1 (Ambra1), a novel ATG gene in the sensitivity or PCa cells to cisplatin. We explored the regulation by the Ambra1 manipulation on the induction of apoptosis and autophagy in human PCa DU145 cells in the presence of cisplatin, via up- or down-regulating Ambra1 expression. In addition, we examined the colony forming of DU145 cells post cisplatin treatment and Ambra1 manipulation. Our results demonstrated that the Ambra1 up-regulation reduced, whereas Ambra1 knockdown increased the cisplatin-induced apoptosis, caspase 3 cleavage, and poly ADP-ribose polymerase (PARP) cleavage. Interestingly, we also found significant autophagy induction in the cisplatin-treated DU145 cells, with increased autophagic vesicles, up-regulated autophagy-related markers. However, the cisplatin-induced autophagy was up-regulated by the Ambra1 overexpression or was down-regulated by the Ambra1 knockdown. In addition, the colony forming was also positively regulated by Ambra1 in DU145 cells post cisplatin treatment. In conclusion, Ambra1 negatively regulates the cisplatin-induced apoptosis and the cisplatin-mediated growth reduction in DU145 cells, in association with the Ambra1-mediated autophagy promotion. It implies that Ambra1-mediated autophagy might be an important mechanism underlining the sensitivity reduction of PCa cells.

## Introduction

Prostate cancer (PCa) is the second most mortal cancer after lung cancer in men from developed countries [1]. Though the androgen deprivation therapy and chemotherapy can control the primary tumor growth in the tumor’s early stages, some patients are resistant (not sensitive) to castration or chemoresistant, needing for more effective treatments [2,3]. Therefore, the treatment options, such as novel antiandrogen or cytotoxic agents are currently needed. In terms of the chemoresistance of PCa, multiple mechanisms have been emphasized. Androgen receptor (AR) axis [4,5], ATP-binding cassette sub-family G member 2 (ABCG2) [6,7] have indicated to promote the drug-resistant activity in human PCa, via several pivotal signaling pathways. A possible critical role of autophagy in cancer onset and progression has recently been underscored by several studies. Multiple autophagy-related proteins have been found to overexpress and to associate with local tumor aggression in the lung, breast, melanoma, and other tumors [8,9]. In PCa, autophagy has also been proposed either as determinant or drug target [10–12].

Cisplatin is a widely used agent for chemotherapy. It can also induce autophagy in various types of cancers, such as endometrial cancer cells [13], malignant mesothelioma cells [14], and cervical cancer HeLa cells [15]. The autophagy induction by cisplatin and its role in chemosensitivity was not well recognized. Activating molecule in beclin1-regulated autophagy1 (Ambra1), a novel ATG gene, has recently been recognized to regulate autophagy [16,17]. Ambra1 regulates Beclin1 [18] or Bcl-2 [16], and then promotes the formation of autophagosomes. Ambra1 may decide the resulting cell death or survival via controlling the conversion between autophagy and apoptosis [19,20]. Moreover, autophagy involves in the cancer therapeutic responsiveness and chemotherapy resistance. Accumulating evidence has revealed that autophagy can be induced in such cancer treatments, as chemotherapy, irradiation, and molecular-targeted therapy [21,22]. Ambra1 has been confirmed to contribute to the cisplatin-resistance in breast cancer cells [23]. Immunohistochemistry and Western blotting results in a previous study demonstrated that an increased expression of AMBRA1 in PCa, positively correlating with the Gleason score [24], implying a possible implication of autophagy in PCa and of AMBRA1 as a possible biomarker in the progression of PCa.

In the present study, we investigated the possible role of Ambra1, a novel ATG gene, on the cisplatin-induced apoptosis and cisplatin-mediated growth reduction in human PCa DU145 cells. Our study found the negative regulation by Ambra1 on the cisplatin-induced apoptosis and cisplatin-mediated growth reduction in DU145 cells, in associating with the Ambra1-mediated autophagy promotion. It implies that Ambra1-mediated autophagy might be an important mechanism underlining the sensitivity reduction of PCa cells.

## Material and methods

### Cell culture and treatment

Human PCa DU145 cells (ATCC, Manassas, VA, U.S.A.) were cultured in RPMI-1640 medium (Invitrogen, Carlsbad, CA, U.S.A.), being supplemented with 10% (v/v) fetal bovine serum (FBS; Invitrogen, Carlsbad, CA, U.S.A.) (or 2% FBS for cell maintaining) in a humidified chamber at 5% CO_2_ at 37°C. DU145 cells were divided when growing to more than 85% confluence. For the Ambra1 overexpression, 85% confluent DU145 cells in six-well (approximately 8 × 10^5^ cells per well) or 12-well plate (approximately 4 × 10^5^ cells per well), without antibiotics, were transfected with Ambra1-pcDNA3.1(+) (with CAT-pcDNA3.1(+) as control) using Lipofectamine 2000 reagent (Invitrogen, Carlsbad, CA, U.S.A.). For the Ambra1 knockdown, 85% confluent cells without antibiotics were transfected with siRNA-Ambra1 or siRNA-Control (final concentration of 50 or 100 nM) using Lipofectamine RNAiMax (Invitrogen, Carlsbad, CA, U.S.A.). For cisplatin treatment, 85% confluent DU145 cells, with Ambra1 overexpressed or knockdown, were treated with 10 μM cisplatin (Sigma-Aldrich, St. Louis, MO, U.S.A.) and were incubated with RPMI-1640 medium/2% FBS in a humidified chamber at 5% CO_2_ at 37°C.

### Quantitative real-time PCR assays for mRNA expression

mRNA sample from DU145 cells was extracted with TRIzol reagent (Life Technologies, Grand Island, NY, U.S.A.) according to the product’s manual. Takara One Step real time PCR (RT-PCR) kit (RR064A, Takara, Tokyo, Japan) was utilized to relatively quantify the Ambra1 mRNA level to β-actin in a 7500 Fast PCR instrument (Applied Biosystems, U.S.A.). The cycle threshold (*C*_t_) values of Ambra1 mRNA were normalized to β-actin from the same sample as relative mRNA levels.

### Western blotting assay

Treated cells were lyzed with ice-cold lysis buffer (Invitrogen, U.S.A.) for 20 min; cell lysate was centrifugated at 13000 ***g*** at 4°C for 30 min, and the supernatant was collected as the total cellular protein extract. Protein samples were successively quantified with BCA Protein Assay Kit (Bio-Rad, Hercules, CA, U.S.A.), were separated with 10% SDS polyacrylamide gel, and then were electrophoretically transferred to nitrocellulose membrane (Millipore, Bedford, MA, U.S.A.). Then the membrane was subject to blocking overnight at 4°C with 5% non-fatty milk in 1× phosphate buffered saline Tween-20 (PBST). Then the membrane was incubated at 4°C for 2 h with rabbit monoclonal antibody against Ambra1, β-actin, caspase 3 (reactive to both pro- and cleaved caspase 3), poly ADP-ribose polymerase (PARP), p62, A and B subunits of microtubule-associated protein1 light chain 3 (LC3-A/B), Atg7, or Beclin1. Finally, the specific binding of antigen and antibody was detected post another incubation at 4°C for 1 h with the secondary HRP-conjugated antibody and the last incubation with enhanced chemiluminescence (Thermo Scientific, Rockford, IL, U.S.A.). Three-time washing with PBST was performed before each incubation. Each band was quantified using Image J software.

### Cell apoptosis assay and caspase 3 activity assay

The apoptosis of DU145 cells was examined by FACScan flow cytometer as following. Post staining with Annexin V-FITC Apoptosis Detection Kit (Abcam, Cambridge, U.K.) according to the guidance of the kit’s manual. Treated or control DU145 cells were trypsinized with 0.25% trypsin (Amresco, Framingham, MA, U.S.A.) and were immediately washed once with ice-cold PBS (by the concentration at 800 ***g*** at 4°C), and then were suspended in the 500–1000 μl 1× binding buffer (1 × 10^6^ cells/ml). Finally, 5–10 μl Annexin V-FITC and propidium iodide (PI) were added on turn into cell suspension for approximately 10 min incubation at room temperature at dark. The flow cyometry analysis was performed on a FACScan flow cytometer (Bio-Rad, Hercules, CA, U.S.A.).

The caspase 3 activity in DU145 cells was examined using Caspase 3 Assay Kit (Colorimetric) (ab39401, Abcam, Cambridge, U.K.). The pellet of 1–5 × 10^6^ cells was resuspended in 50 µl of chilled Cell Lysis Buffer and was incubated on ice for 10 min. The cell lysate was centrifuged at 10000 ***g*** for 5 min, and the supernatant was transferred to tube on ice. The protein concentration was measured with BCA Protein Assay Kit (Bio-Rad, Hercules, CA, U.S.A.), and was adjusted to 50–200 µg per 50 µl Cell Lysis Buffer for each assay (well). Then each cell well was added with 50 µl of 2× Reaction Buffer (containing 10 mM DTT) and with 5 μl of the 4 mM DEVD-p-NA substrate (200 μM final concentration) for an incubation at 37°C for 60–120 min. Then the plate was measured for the OD400-405 nm on a microplate reader (Bio-Rad Laboratories Inc., Hercules, CA, U.S.A.). The caspase 3 activity was presented as a relative level to control.

### Quantitative analysis of autophagic vesicles with GFP-LC3B reporter

The autophagic vesicles in DU145 cells was visualized and quantified with a green fluorescence protein (GFP)-LC3B reporter, pGM-CMV-GFP-hLC3 (Genomeditech, Shanghai, China). Briefly, approximately 85% confluent DU145 cells were transfected with the pGM-CMV-GFP-hLC3 reporter plasmid with Lipofectamine 2000 (Invitrogen, Carlsbad, CA, U.S.A.). Post an incubation for 24 h, DU145 cells were updated with RPMI-1640 medium supplemented with 2% FBS. Then the GFP-positive vesicles were visualized and counted under Hitachi Medical Systems Fluorescence F-2000 (Hitachi, Tokyo, Japan).

### Colony forming assay

The regulation by cisplatin, or by the Ambra1 manipulation on the growth of DU145 cells was evaluated by colony forming assay. In brief, 200-300 blank DU145 cells or the DU145 cells post Ambra1 up- or down-regulation were seeded in six-well plates and were incubated for 24 h, at 37°C under 5% CO_2_. Then cells were treated with 10 μM cisplatin for another 24 h. Post another incubation for 5–8 days at 37°C under 5% CO_2_, the DU145-formed colonies were stained Crystal Violet (0.005%) for 20 min and were counted.

### Statistical evaluations

Results are presented as mean ± standard error of the mean (SE). For the analysis between two groups, the Student’s *t* test was performed. A *P* value of 0.05 or much less was considered significant.

## Results

### Ambra1 blocks the cisplatin-induced apoptosis in human PCa DU145 cells

**Figure 1:**
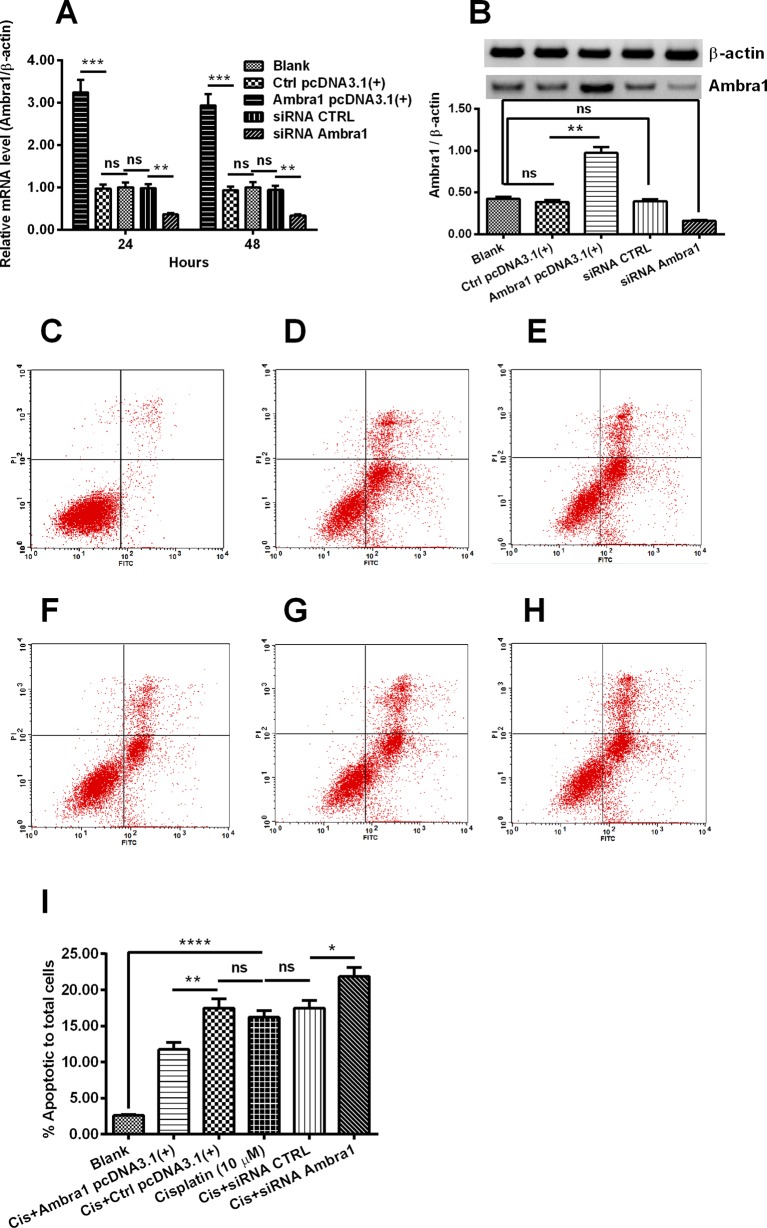
Ambra1 manipulation and its regulation on of the apoptosis in the DU145 cells post cisplatin treatment (**A** and **B**) Relative Ambra1 mRNA level (A) or Western blot analysis of Ambra1 (B) in blank DU145 cells, or in the DU145 cells, which were transfected with CAT-pcDNA3.1(+) (Ctrl pcDNA3.1(+)), with Ambra1 pcDNA3.1(+), with Control siRNA (siRNA CTRL), or with Ambra1-specific siRNA ((siRNA Ambra1) for 24 h (Ambra1 mRNA level) or 48 h (both mRNA and protein levels of Ambra1). (**C**–**I)** FACS analysis (C–H) or quantification (I) of apoptotic cells in blank group (C), in the cisplatin-treated (10 μM) DU145 cells (D), in the up-regulation control DU145 cells (Cis+Ctrl pcDNA3.1(+)) (D) or in the Ambra1-overexpressed DU145 cells (Cis+Ambra1 pcDNA3.1(+)) (E), in the down-regulation control (Cis+siRNA CTRL) (F) or in the Ambra1-knocked down DU145 cells (Cis+siRNA Ambra1) (H). Early and later apoptotic cells were denoted respectively in the lower right quadrant and upper right quadrant. Results were presented as mean ± standard error of the mean (SEM) (*n* = 3 for each group); *P<0.05, **P<0.01, ***P<0.001, or ****P<0.001, ns: no significance.

To investigate the regulatory role of Ambra1 in the cisplatin-induced apoptosis in DU145 cells, we manipulated the Ambra1 expression in DU145 cells, and then evaluated the apoptosis in DU145 cells, post cisplatin treatment. As indicated in Figure 1, the transfection with Ambra1-pcDNA3.1(+)) significantly up-regulated the mRNA level of Ambra1 in DU145 cells (*P*<0.001) (Ambra1 pcDNA3.1(+), first column), compared with the control group of DU145 cells, which were transfected with CAT-pcDNA3.1(+) (Ctrl pcDNA3.1(+), second column) at either 24- or 48-h post transfection. The Ambra1 mRNA promotion lasted from 24 to 48 h, though there was unrecognizable reduction of it at 48-h post transfection. Of course, there was no significant difference in the mRNA level of Ambra1 between CAT-pcDNA3.1(+)-transfected and blank (third column) DU145 cells. In the other experiment, we knocked down the Ambra1 expression with RNAi technology. In contrast with the siRNA-Ctrl (control)-transfected DU145 cells (siRNA CTRL, column 4), the transfection with siRNA-Ambra1 markedly reduced Ambra1 expression in DU145 cells (siRNA Ambra1, column 5) (*P*<0.01). The up-regulation by Ambra1-pcDNA3.1(+) or the down-regulation with siRNA-Ambra1 was also confirmed in protein level by Western blotting assay. Figure 1B indicated a higher or a less protein level of Ambra1 in the Ambra1-pcDNA3.1(+)-transfected or in the siRNA-Ambra1-transfected DU145 cells (*P*<0.01 respectively).

Then the DU145 cells, with Ambra1 overexpressing or knocking down were treated with 0 or 10 μM cisplatin for 48 h, and the apoptosis was assessed by flow cytometry post Annexin V-FITC/PI staining. Figure 1C demonstrated that there were only approximately 3% apoptotic cells in the blank DU145 cell group. However, the apoptosis was markedly induced by the treatment with 10 μM cisplatin to as high as about 17% in DU145 cells (*P*<0.0001, Figure 1D). Although CAT-pcDNA3.1(+) did not significantly regulated the cisplatin-induced apoptosis (no significant compared with the cisplatin-treated DU145 cells) (Figure 1E), the Ambra1-pcDNA3.1(+) transfection markedly blocked the cisplatin-induced apoptosis in DU145 cells (*P*<0.01), reducing the apoptosis induction to approximately 12% (Figure 1F). On the other side, the transfection with siRNA-Ambra1 significantly aggravated the apoptosis induction to more than 20% (Figure 1G), compared with the transfection with siRNA-Ctrl (Figure 1H) (*P*<0.05). Detailed counting of apoptotic DU145 cells was indicated in Figure 1I.

**Figure 2:**
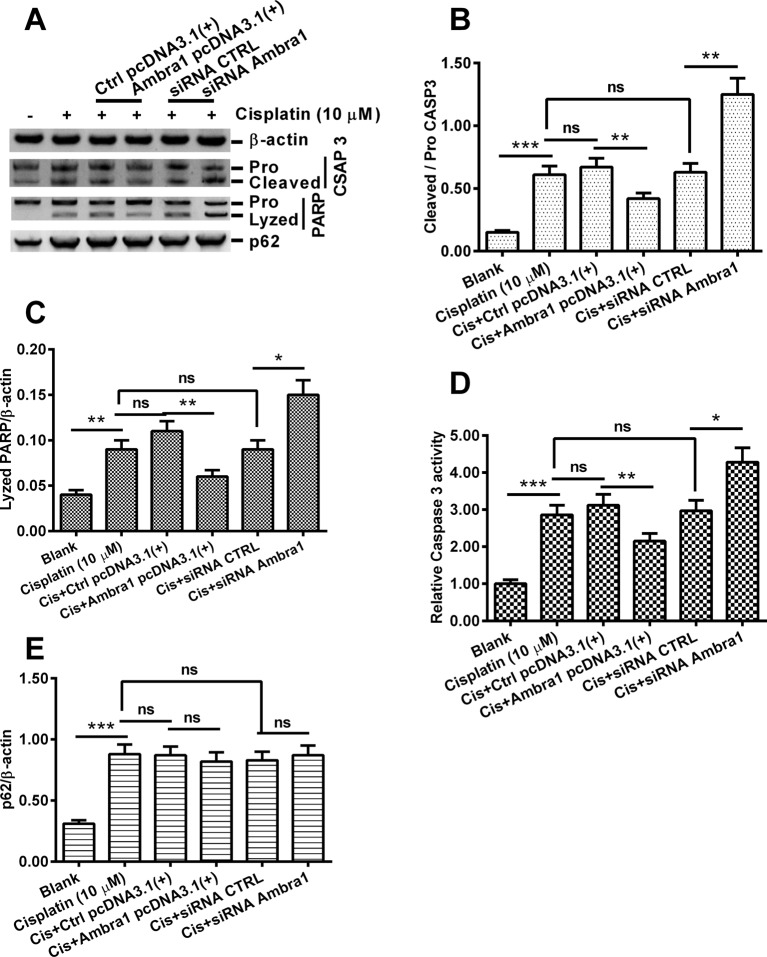
Western blot analysis of apoptosis-associated proteins in the cisplatin-treatment DU145 cells post Ambra1 manipulation (**A**) Western blot analysis of caspase 3 cleavage (pro-CASP 3 to cleaved CASP 3) and PARP (poly ADP-ribose polymerase) lysis in the blank, cisplatin (10 μM), Cis+Ctrl pcDNA3.1(+), Cis+Ambra1 pcDNA3.1(+), Cis+siRNA CTRL, or Cis+siRNA Ambra1 DU145 cells. (**B** and **C**) Ratio of cleaved CASP 3 (B) or lyzed PARP (C) to β-actin in above-mentioned groups DU145 cells; (**D**) Relative caspase 3 activity in above-mentioned groups of DU145 cells.(**E**) Ratio of p62 to β-actin in above-mentioned groups DU145 cells; Results were presented as mean ± standard error of the mean (SEM) (*n* = 3 for each group); *P<0.05, **P<0.01, or ***P<0.001, ns: no significance.

Western blotting assay (Figure 2A) was also performed for apoptosis-associated biomarkers (cleaved caspase 3 and PARP) in the cisplatin-treated DU145 cells, with Ambra1 overexpressed or knocked down. Figure 2B demonstrated that the cisplatin-induced significantly high level of caspase 3 cleavage (*P*<0.001, compared with blank cells) was markedly reduced in the Ambra1 pcDNA3.1(+) DU145 cells (*P*<0.01, compared with Ctrl pcDNA3.1(+)). On the other side, it was significantly aggravated in the siRNA Ambra1 DU145 cells, compared with siRNA CTRL cells (*P*<0.01). Similar regulatory effect was also found in the lysis of PARP (Figure 2C). In addition, we also examined the caspase 3 activity in above-mentioned groups of DU145 cells. The cisplatin-induced significant caspase 3 activity was inhibited or aggravated in Ambra1 pcDNA3.1(+) or in siRNA Ambra1 DU145 cells. In addition, Western blotting of p62 protein level demonstrated that a significant higher level of p62 was induced by cisplatin treatment (*P*<0.001, first vs second column, Figure 2E). However, the Ambra1-pcDNA3.1(+) transfection or the siRNA Ambra1 transfection did not regulate the cisplatin-induced p62 markedly (Figure 2E). Taken together, our results found the negative regulation by Ambra1 on the cisplatin-induced apoptosis in DU145 cells, regardless of p62 signaling pathway.

### Ambra1 promotes the autophagy in the cisplatin-treated DU145 cells

**Figure 3:**
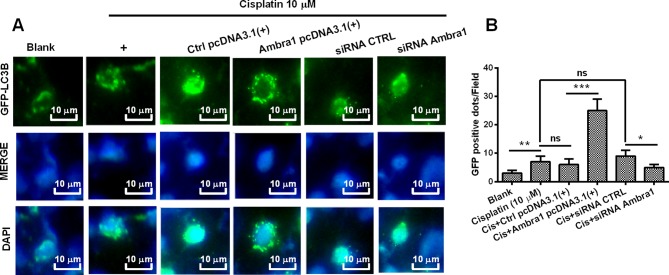
Autophagic puncta in the cisplatin-treatment DU145 cells post Ambra1 manipulation (**A**) Autophagic vesicles (acidic vesicular organelles, AVOs) in the DU145 cells, which were treated with 0 (Blank), or cisplatin-treated DU145 cells (10 μM), or in the Cis+Ctrl pcDNA3.1(+), Cis+Ambra1 pcDNA3.1(+), Cis+siRNA CTRL, or Cis+siRNA Ambra1 DU145 cells. AVOs were shown as the green fluorescence protein (GFP)-positive puncta in DU145 cells, which were transfected with LC3B-GFP reporter plasmid. (**B**) Quantification of autophagic vesicles in the above-mentioned DU145 cells. Images were obtained by confocal microscopy in triple independent replicate experiments; *P<0.05, **P<0.01, ***P<0.001, ns: no significance.

Acidic vesicular organelles (AVOs) symbolize autophagy formation [25]. The microphotographs of AVOs were observable under fluorescence microscope in target cells post the transfection with the GFP-LC3 report vector. As shown in Figure 3A, compared with the blank cells (first column), there were significantly more GFP-positive AVOs (GFP-positive dots) in the cisplatin-treated (10 μM) DU145 cells (second column). Compared with the Ctrl pcDNA3.1(+) DU145 cells (third column, Figure 3A), there were even more GFP-positive AVOs in the Ambra1-overexpressed (Ambra1 pcDNA3.1(+)) DU145 cells (third column, Figure 3B). On contrary, in contrast with in the siRNA CTRL DU145 cells (fourth column, Figure 3B), there were less AVOs in the siRNA Ambra1 cells (fifth column, Figure 3B). The significant difference was quantified in Figure 3B (*P*<0.05, *P*<0.01, or *P*<0.001).

**Figure 4:**
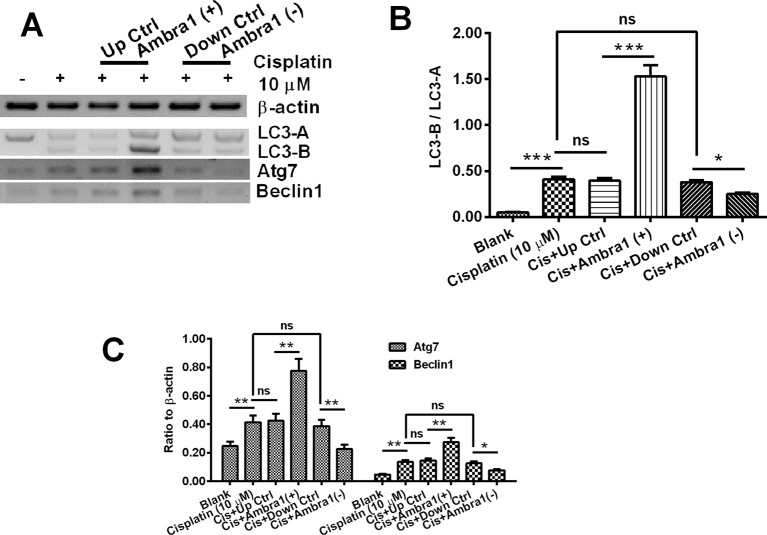
Western blot analysis of autophagy-associated proteins in the cisplatin-treatment DU145 cells post Ambra1 manipulation (**A**) Western blot analysis of the conversion of LC3-A to LC3-B, the expression of Atg7 and Beclin1 in the blank, cisplatin (10 μM), Cis+Ctrl pcDNA3.1(+), Cis+Ambra1 pcDNA3.1(+), Cis+siRNA CTRL, or Cis+siRNA Ambra1 DU145 cells. (**B** and **C**) Ratio of LC-3B/LC3-A (B) or the relative level of Atg7 and Beclin1 to β-actin (C) in above-mentioned groups DU145 cells. Results were presented as mean ± standard error of the mean (SEM) (*n* = 3 for each group); *P<0.05, **P<0.01, or ***P<0.001, ns: no significance.

Western blotting was also performed to examine the expression of Atgs, such as LC3, Atg7, and Beclin1. Figure 4A demonstrated that the conversion of LC3-A to LC3-B, which is the marker of autophagy [26], was significantly up-regulated in the cisplatin-treated DU145 cells (*P*<0.001, Figure 4B), and the conversion was even more higher in the Ambra1 pcDNA3.1(+) DU145 cells than in the Ctrl pcDNA3.1(+) DU145 cells (*P*<0.001, Figure 4B). On the other side, there was less LC3-B/LC3-A in the siRNA Ambra1 DU145 cells than in the siRNA CTRL DU145 cells (*P*<0.05, Figure 4B). Figure 4C demonstrated similar regulation by Ambra1 on the expression of Atg7 and Beclin1. The cisplatin-promoted Atg7 and Beclin1 were aggravated in Ambra1 pcDNA3.1(+) DU145 cells, whereas were reduced in siRNA Ambra1 DU145 cells (*P*<0.05 or *P*<0.01). Taken together, we confirmed the promotion by Ambra1 on the autophagy in the cisplatin-treated DU145 cells.

### Ambra1 ameliorates the growth of cisplatin-treated DU145 cells

**Figure 5:**
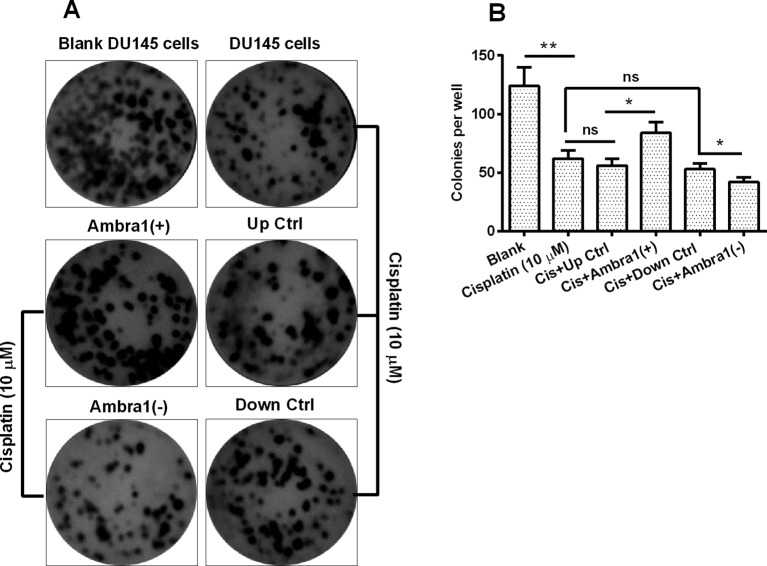
Regulation by Ambra1 on the cisplatin-medicated colony forming reduction of DU145 cells Representative images (**A**) and quantification (**B**) of the colony formation by the blank DU145, cisplatin-treated (10 μM), Cis+Ctrl pcDNA3.1(+), Cis+Ambra1 pcDNA3.1(+), Cis+siRNA CTRL, or Cis+siRNA Ambra1 DU145 cells. Each quantitative data was averaged for triple independent results. Statistical significance was shown as *P<0.05, **P<0.01, ns: no significance.

We also explored the regulation by Ambra1 on the growth of cisplatin-treated DU145 cells with colony forming assay. It was shown in Figure 5A that the cisplatin treatment markedly reduced the du145-formed colonies (*P*<0.01, column 2 vs column 1, Figure 5B). However, such reduction was ameliorated by the Ambra1 overexpression, indicating more colonies in Ambra1 pcDNA3.1(+) group than in Ctrl pcDNA3.1(+) group (*P*<0.05, column 4 vs column 3, Figure 5B); and the Ambra1 knockdown significantly deteriorated such reduction, indicating less colonies in siRNA Ambra1 group than in siRNA CTRL group (*P*<0.05, column 6 vs column 5). Therefore, Ambra1 ameliorates the growth of cisplatin-treated DU145 cells.

## Discussion

Ambra1 has recently been recognized to promote the formation of autophagosomes and thus regulate autophagy [16,17]. It was speculated that Ambra1 might control the cell death or survival via converting autophagy and apoptosis [19,20]. Increased expression of Ambra1 has been implicated in PCa [24], implying a possible implication of autophagy in the progression of PCa. In the present study, we found the blockage by Ambra1 to the cisplatin-induced apoptosis in human PCa DU145 cells. Results both from flow cytometry analysis for apoptotic cells and from Western blot analysis for apoptosis-associated biomarkers (cleaved caspase 3 and PARP) supported the inhibition by Ambra1 overexpression to the cisplatin-mediated apoptosis induction in PCa DU145 cells. On the other side, our results also supported the conclusion that the siRNA-mediated knockdown of Ambra1 aggravated the cisplatin-induced apoptosis in DU145 cells. Therefore, our results found the negative regulation by Ambra1 on the cisplatin-induced apoptosis in PCa DU145 cells.

Considering the important role of autophagy in the regulation on cell survival and cell death, it is critical for evaluating autophagy as a cancer therapy strategy to understand the signaling pathways underlying autophagy in response to various conditions in cancer. The autophagosome elongation-related Atgs, such as Atg5 [11], P21 [27], UNC-51-like kinase1 (ULK1) [28] have been suggested in PCa. We hypothesized that autophagy might be the mechanism underlining the negative regulation by Ambra1 on the cisplatin-induced apoptosis in DU145 cells. Our further results confirmed the promotion by Ambra1 to autophagy in the cisplatin-treated DU145 cells. Results from the autophagy-symbolized AVOs [25], the conversion of LC-3A to LC-3B, and the expression of Atg7 and Beclin1 unanimously confirmed the positive regulation by Ambra1 on the autophagy in the cisplatin-treated DU145 cells. The positive regulation by Ambra1 on autophagy was also supported by the results that the autophagy was inhibited by Ambra1 knockdown in the cisplatin-treated DU145 cells. Therefore, there was an association between the Ambra1-mediated positive regulation on autophagy and the Ambra1-mediated negative regulation on apoptosis.

Accumulating evidence has revealed that autophagy involves in the cancer therapeutic responsiveness [21,22]. Autophagy inhibition might sensitize PCa cells to proapoptotic stimuli. Indeed, it has been shown that autophagy blockage *in vitro* sensitizes PCa cells to Src tyrosine kinase inhibitors [12]. Our study recognized the negative regulation by Ambra1 on the sensitivity of DU145 cells to cisplatin both via the apoptosis analysis and the colony forming assay. The cisplatin-mediated reduction of colony forming was blocked or aggravated by the Ambra1 overexpression or by the Ambra1 knockdown. Generally, the autophagy-mediated protective response to chemotherapy or to radiation in cancer cells implies the potential of autophagy as a possible clinical strategy to counteract therapeutic resistance in PCa [29]. In addition, we did not found a significant regulation by Ambra1 manipulation on p62 protein in the cisplatin-treated cells. Previous study confirmed the regardlessness of p62 in the Ambra1-induced autophagy in human embryonic kidney HEK293 and HeLa cells [30]. Our results confirmed such regardlessness of p62 in the Ambra1-induced autophagy and desensitization to cisplatin in human prostate cancer cells. Therefore, it is reasonable to long for the potential of an Ambra1 inhibitor against the PCa chemoresistance.

In summary, our study found the negative regulation by Ambra1 on the cisplatin-induced apoptosis and cisplatin-mediated growth reduction in DU145 cells, such negative regulation was associated with the Ambra1-mediated autophagy promotion, regardless of p62 signaling pathway. It implies that Ambra1-mediated autophagy might be an important mechanism underlining the sensitivity reduction of PCa cells.

## Abbreviations

ABCG2: ATP-binding cassette sub-family G member 2
Ambra1: activating molecule in beclin1-regulated autophagy1
AR: androgen receptor
AVO: acidic vesicular organelle
PARP: poly ADP-ribose polymerase
PCa: prostate cancer
